# Virtual-reality-enhanced mannequin to train emergency physicians to examine dizzy patients using the HINTS method

**DOI:** 10.3389/fneur.2023.1335121

**Published:** 2024-01-05

**Authors:** Guillaume Ursat, Morgane Corda, Julien Ryard, Christophe Guillet, Caroline Guigou, Cindy Tissier, Alexis Bozorg Grayeli

**Affiliations:** ^1^Emergency Department, Dijon University Hospital, Dijon, France; ^2^Otolaryngology Department, Dijon University Hospital, Dijon, France; ^3^Institut Image, Ecole Nationale d’Arts-et-Métiers, Chalon-sur-Saône, France; ^4^ICMUB, CNRS, Université Bourgogne-Franche-Comté, Dijon, France

**Keywords:** vertigo, acute vestibular syndrome, head-impulse test, HINTS, simulation, training, virtual reality

## Abstract

**Introduction:**

Acute vertigo is a frequent chief complaint in the emergency departments, and its efficient management requires thorough training. The HINTS protocol is a valid method to screen patients in the emergency room, but its application in routine is hindered by the lack of training. This study aimed to evaluate the training of emergency physicians for the HINTS method based on a mannequin-based virtual reality simulator (MBVRS).

**Methods:**

We conducted a monocenter, prospective, longitudinal, and randomized cohort study in an Emergency Department at a regional university hospital. We included 34 emergency physicians randomized into two equal groups matched by age and professional experience. The control group attended a theoretical lesson with video demonstrations and the test group received a simulation-based training in addition to the lecture.

**Results:**

We showed that the test group had a higher diagnosis performance for the HINTS method compared to the control group as evaluated by the simulator at 1 month (89% sensitivity versus 45, and 100% specificity versus 86% respectively, *p* < 001, Fisher’s exact test). Evaluation at 6 months showed a similar advantage to the test group.

**Discussion:**

The MBVRS is a useful pedagogic tool for the HINTS protocol in the emergency department. The advantage of a unique training session can be measured up to 6 months after the lesson.

## 1 Introduction

Acute vertigo accounts for 2% of all emergency visits (1), and this number is steadily increasing (2). Vertigo is indicative of multiple affections ranging from benign inner ear diseases to life-threatening conditions such as a stroke or a central nervous system tumor (3, 4). A rapid, sensitive, and specific diagnosis is crucial to orient the patient to an intensive care unit in case of a neurovascular condition (25% of patients) (5) or toward a symptomatic treatment if an inner ear disorder is diagnosed (6).

No specific clinical sign can definitively distinguish peripheral causes of acute vertigo from central ones (7). It has long been admitted that a cranial MRI with diffusion sequences is necessary and sufficient to rule out a stroke. However, diffusion MRI can be falsely negative in up to 12% of posterior circulation strokes if performed early (8). Moreover, MRI scans are not rapidly accessible in all emergency departments.

To address this issue, Kattah et al. (8) proposed a diagnostic tool consisting of three bedside clinical assessments to distinguish peripheral causes of acute vertigo from central ones. This evaluation includes the analysis of the nystagmus (central or peripheral type), the detection of a skew deviation (indicative of a supranuclear disorder), and the head-impulse test (HIT or Halmagyi–Curthoys test, in which the catch-up saccade reveals the side of the affected inner ear). This diagnosis tool is called HINTS, standing for Head Impulse, Nystagmus, and Test of Skew.

A 2-min bedside clinical evaluation using the HINTS method in patients with acute vestibular syndrome and without apparent neurological and otological abnormalities who present at least one cardiovascular risk factor is more efficient than MRI and yields a 100% sensitivity and 96% specificity when it is performed by a trained clinician (9, 10). This method has also shown its superiority to other clinical tests such as ABCD2 used in stroke evaluations (11, 12). This tool is of major interest to the public health sector as it leads to rapid and effective case management and prevents excessive imaging prescriptions and unnecessary diagnostic workups.

The optimal application of the HINTS method in real-life conditions requires significant theoretical and practical training. Not only theoretical courses on the clinical examination of a dizzy patient are inconsistent in emergency departments but also practical training in real conditions is long and difficult to organize; thus, there is a serious lack of support for emergency physicians regarding transmission of knowledge about management of patients with dizziness. Moreover, the HINTS method is effective but requires practical skills that emergency clinicians may not develop through theory alone. This highlights the need for better hands-on training in emergency care settings.

Alternatives for physicians’ training have been attempted in the past, such as the use of a mannequin to train emergency residents for the head impulse test stated by Omron et al. (13). Although the results have not been published, this abstract gives us the first hint on the potential added value of using external tools to raise both knowledge and confidence (or comfort) in emergency physicians’ diagnosis regarding central or peripheral causes of vertigos. Another training tool for the head impulse test was developed by MacDougall et al. in 2012 [aVOR (iPhone and iPad App). 1.1 ed. Apple App Store: Liberty information technology] and used for training and understanding of virtual head impulse test (14). This app represents a great theoretical work support for physicians to understand the cause-and-effect relationship of semi-circular deficit and impact on catch-up saccade but does not allow manipulation training.

New tools were needed not only to train physicians on all three parts of the HINTS examination but also on patient handling. For this purpose, a pedagogical simulator using a mannequin-based virtual reality simulator (MBVRS) was developed for the clinical examination of patients with vertigo (15).

The value of practical training in the emergency department (16, 17) and the development of virtual reality (18–21) as a training tool have been widely documented. To the best of our knowledge, there is no other virtual-reality simulation tool that offers training possibilities for the clinical examination of vertiginous patients based on the HINTS method.

The aim of this study was to assess the value of this MBVRS to train emergency physicians for the clinical examination of dizzy patients using the HINTS method. The study was performed in an emergency department at a regional university hospital.

## 2 Materials and methods

We conducted a monocenter, prospective, longitudinal, and randomized cohort study in an emergency department at a regional university hospital to assess the effect of simulation training on diagnostic performances using the HINTS method. Seventeen physicians were trained with the simulator and 17 controls only received a theoretical lesson. This protocol was reviewed and approved by the institutional ethical committee (CCP Est I).

### 2.1 Study design

**Figure 1:**
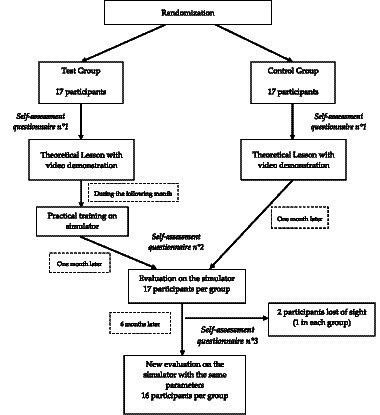
Study design.

The study was conducted from May to November 2022 (Figure 1). All emergency physicians and residents in the emergency department were contacted (*n* = 40). They all received information about the study objectives, its sequence, and the use of the pedagogical simulator. Among these, 34 volunteers (90%) were included. All the participants attended a 45-min lecture on the HINTS protocol, which included videos and pictures and provided explanations on the execution of each step and the semiology of each subtest followed by 15 min of free questions.

The population was divided into two randomized groups of 17 practitioners (test and control), matched by their professional experience (5 years or more and less than 5 years and residents).

One month after the lesson, participants in the test group attended 1 h of practical training with the simulator, supervised by the same emergency physician and otorhinolaryngologist for all participants. The control group did not receive any other training on the subject.

The test group was assessed on the simulator 1 month after the practical training. The control group was also evaluated 1 month after the theoretical lesson in the same conditions as the test group. Both groups were evaluated again 6 months after the practical training (test group) or the theoretical lesson (control group). For the control group, an additional 15-min familiarization time was allowed before the tests.

Before the theoretical lesson and the two evaluations, participants answered auto-questionnaires and rated their knowledge and practical experience with the HINTS method (Likert score from 0 to 10) and their overall knowledge and confidence level in their clinical examination of a dizzy patient (Likert score from 0 to 10), as well as an open question on situations that seem to require HINTS evaluation.

The self-assessment questionnaires contained the following questions:

- Inclusion

1. Professional experience: Single choice response: Undergraduate/ Graduated <5 years/Graduated ≥5
2. Do you know the HINTS? Yes/No
3. If yes, do you practice it? Yes/No
4. On a scale of 0 to 10, how would you rate your theoretical knowledge acute vertigo management in the Emergency ward? Likert scale
5. On a scale of 0 to 10, how would you rate your practical knowledge acute vertigo management in the Emergency ward? Likert scale
6. On a scale of 0 to 10, how much do you trust your clinical findings when examining a patient with acute vertigo? Likert scale
7. How often do you prescribe brain imaging in the context of an acute vertigo in the ER? Single choice response: Always/> 50% of cases/< 50% of cases/Never
8. If you suspect a peripheral vertigo, how often will you prescribe a brain imaging to confirm your hypothesis?
9. Single choice response: Always/Never/Depending on the context
10. If according to the context, what are the criteria that lead you to prescribe a brain imaging? (Open question)

II One- and 6-month follow-up questionnaires

1. Since the beginning of the study, how often did you use the HINTS method on patients with an acute vertigo in the ER?
2. Single choice response: Always/> 50% of cases/< 50% of cases/Never/I did not have the chance to
3. Today, on a scale of 0 to 10, how would you rate your theoretical knowledge acute vertigo management in the Emergency ward? Likert scale
4. Today, on a scale of 0 to 10, how would you rate your practical knowledge acute vertigo management in the Emergency ward? Likert scale
5. Currently, on a scale of 0 to 10, how much do you trust your clinical findings when examining a patient with acute vertigo? Likert scale
6. Since the beginning of the study, how often have you prescribed brain imaging in the context of an acute vertigo in the ER?
7. Single choice response: Always/> 50% of cases/< 50% of cases/Never/I did not have the chance
8. Since the beginning of the study, how often did you prescribe a brain imaging to confirm your diagnostic hypothesis of a peripheral acute vertigo?
9. Single choice response: Always/Never/Depending on the context
10. If according to the context, what are the criteria that lead you to prescribe a brain imaging? (Open question)
11. Two participants, one in each group, were lost to follow-up at 6 months.

### 2.2 The simulator

**Figure 2:**
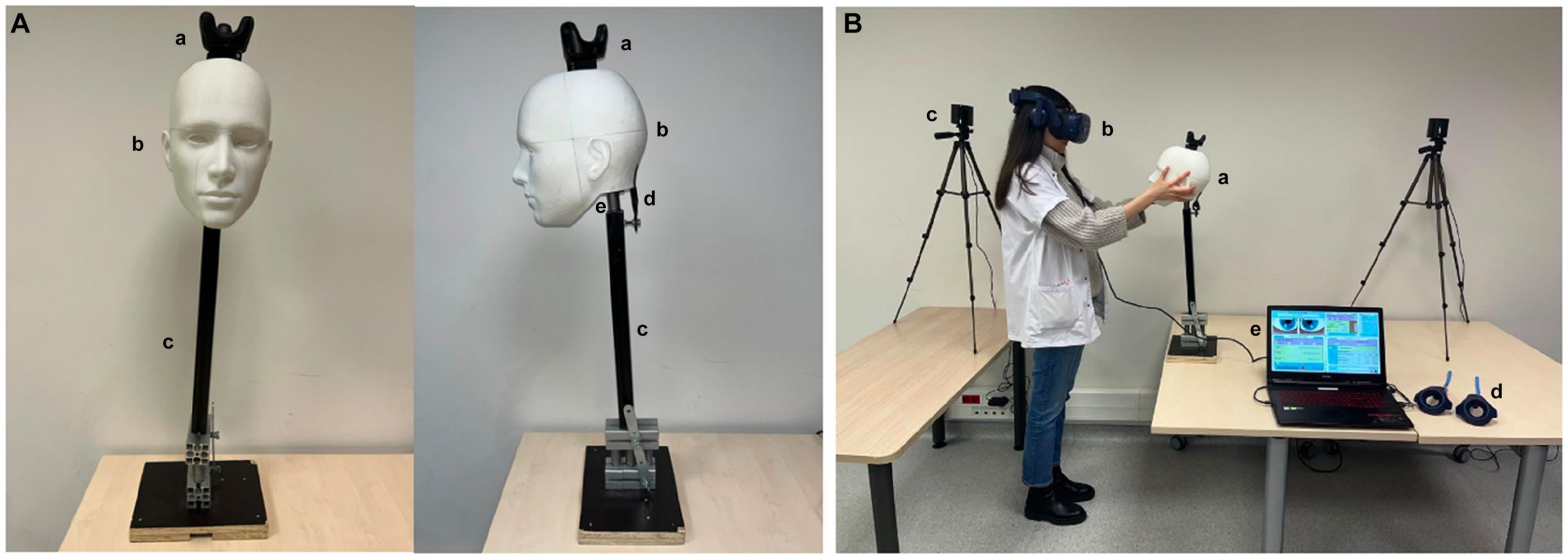
Virtual-reality-enhanced mannequin simulation system. **(A)** The mannequin is composed of a 3D-printed true size human head **(a)**, **(b)** a stiff rod, **(c)** and a base that can be settled down on a table. An elastic **(d)** and a spring **(e)** allow head movements during examination. The mannequin’s head has a tracker for software analysis. **(B)** The learner faces the mannequin **(a)** wearing the virtual reality helmet **(b)**. The cameras, **(c)** to detect the controllers, and **(d)** mannequin and headset position. The controllers **(d)** are used to calibrate the system and achieve ocular monitoring for nystagmus and skew deviation analysis. The trainer programs the different scenarios on the laptop computer **(e)**.

**Figure 3:**
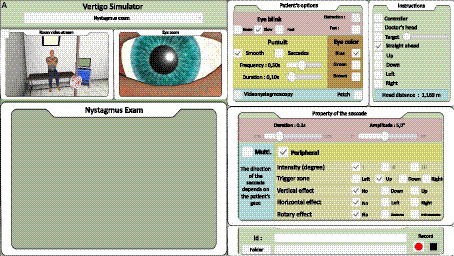
Trainer interface: for nystagmus **(A)**. Skew deviation and head impulse test analysis interface are shown in Supplementary Data.

A pedagogical MBVRS of a dizzy patient (VertImage) was developed (15). The device is composed of a virtual reality (VR) headset (HTC Vive Pro ®, HTC Corp., Taoyuan, Taiwan), two tracking cameras, a laptop, an articulated mannequin head bearing a tracker on its vertex, with two controllers for calibration purposes, serving as a red target to examine the avatar’s ocular movements, and as a cover to assess the skew deviation (Figure 2). The trainee wore the VR headset and was immersed in a medical examination room in front of a patient seated on an examination table (Figure 2). The avatar’s head position, size, and form corresponded to an articulated mannequin head [three-dimensional (3D) printing] articulated on a stiff rod with a spring and an elastic band to reproduce flexible cervical movements. The mannequin faced the learner. The system provided the simulation possibilities for the three subtests of the HINTS protocol: nystagmus, HIT, and skew deviation (Figure 3). For the HIT, the mannequin head-impulse was detected by the system due to the tracker fixed on the mannequin’s vertex, and the avatar’s eyes moved according to the selected parameters by the trainer. For the nystagmus analysis and skew deviation, the controllers served as moving targets or covers in front of the avatar’s eyes.

The software was developed on a Unity3D platform (Unity Technologies, San Francisco, CA). The tracker position was sampled at 60 Hz. The delay between the mannequin’s head movements and avatar movements was estimated at 58 ms including the transmission time between the tracker movement and the software processing (6 ms), the avatar’s position estimation by the software (11 ms), and the Unity3D reaction delay (31 ms). Avatar’s movements were sampled at 120 Hz for the ocular movements. The display (headset) refresh rate was 90 Hz. The system ran on a laptop computer (Intel® CoreTMI7-8750H CPU at 2.2 GHz, and 16.0 GB RAM) including a graphic processing unit (GPU, NVIDIA GeForce RTX 2060) and 6 GB dedicated RAM.

The trainer had a monitoring screen (laptop, Figure 3) on which the participant could observe the virtual scene and select standardized scenarios for training and evaluation purposes as follows:

• HIT
– Fixed parameters: saccade duration = 25 ms, irregular blink average frequency = 0.3 Hz.
– Seven scenarios: no deficit, lateral semicircular canal deficits with variable catch-up saccade delays (left and right deficits with 80, 200, and 320 ms saccade delays).
– A correct answer was noted when the trainee correctly executed the maneuver, identified the central or peripheral origin of the sign, and indicated the correct side of the deficit.
• Nystagmus
– Fixed parameters: irregular blink average frequency = 0.3 Hz, nystagmus frequency = 2 Hz, and nystagmus angular amplitude = 5°.
– Ten scenarios: left and right horizontal peripheral nystagmus with a torsional component and 3 intensity grades (1: present only in lateral gaze to the side of the nystagmus, 2: present in lateral gaze and straight-ahead gaze, and 3: present in all eye positions), one case of central vertical downward nystagmus on downward gaze, one case of central multi-directional nystagmus and two situations with no nystagmus.
– A correct answer was noted when the trainee identified the central or peripheral type and the correct nystagmus direction.
• Skew deviation
– Stable parameters: irregular blink average frequency = 0.3 Hz.
– Five scenarios: 1.5° downward on the right eye, 3° downward on the left eye, 4.5° downward on the left eye, and two situations without deviation.
– A correct answer was noted when the trainee oriented the sign to the central (skew deviation present) or peripheral (no skew deviation) origin of the sign. It has been shown that skew deviation could hardly be present in acute vestibular syndromes (22), but it is considered an argument if present during the HINTS test.

All the scenarios were randomly proposed to all the participants for training and the two evaluation sessions at 1 and 6 months.

### 2.3 Primary and secondary outcome criteria

The primary outcome criterion was an increase of the HINTS sensitivity and specificity in the test group at 1 month after VR practical training.

Secondary outcome criteria included an increase of the HINTS sensitivity and specificity in the test group at 6 months and higher overall knowledge and confidence levels in clinical examination in the test group as judged by the questionnaire scores at 1 and 6 months.

### 2.4 Statistical analysis

Study criteria were determined *a priori* with an alpha risk of 20% and statistical power of 80%. The number of subjects required was estimated with G*Power software (23).

To determine the number of subjects required, we hypothesized that the answers would be randomly given in the worst case (50% of true positives in this situation). The results would be considered significant if 90% of true positives were found within the test group. The inclusion of 16 participants in each group yielded a 0.82 power and an alpha risk of 0.11.

An exact Fisher’s test was employed to compare the percentages of the correct answers. We used the Mann–Whitney tests to compare Likert’s scores between groups, and the Wilcoxon test was used to compare scores within the same group at different stages of the study. A value of *p* of <0.05 was considered statistically significant.

## 3 Results

### 3.1 Participants

The sex ratio (male/female) was 11/6 in both groups. Six participants in each group graduated after more than 5 years (35%), and six participants (35%) in the group test graduated in less than 5 years versus five participants (29%) in the control group. Five participants (29%) in the test group and six (35%) in the control group were residents.

### 3.2 Diagnostic performance

Emergency physicians trained with VertImage showed higher diagnostic performances with the HINTS method compared to the control group, at both 1-month and 6-month endpoints (Tables 1, 2).

**Table 1:** Performances of the test (simulator) and control groups at 1 month.

**Group**	**Hit**				**Nystagmus**				**Skew**				**Total**			
	TP	FN	Se	Value of *p*	TP	FN	Se	Value of *p*	TP	FN	Se	Value of *p*	TP	FN	Se	Value of *p*
Test	92	10	90.2	<0.0001	117	19	86.0	<0.0001	47	4	92.2	0.0007	256	33	88.6	<0.0001
Control	33	69	32.4		64	72	47.1		32	19	62.8		129	160	44.6	
	TN	FP	Sp		TN	FP	Sp		TN	FP	Sp		TN	FP	Sp	
Test	17	0	100.0	0.0009	34	0	100.0	>0.9999	34	0	100.00	0.4925	85	0	100.0	0.0003
CONTROL	8	9	47.1		33	1	97.1		32	2	94.12		73	12	85.9	

TP, true positives; FN, false negatives; TN, true negatives; FP, false positives; Se, sensitivity; Sp, specificity. Value of *p*s correspond to Fisher’s exact tests.

**Table 2:** Performances of the test (simulator) and control groups at 6 months.

**Group**	**Hit**				**Nystagmus**				**Skew**				**Total**			
	TP	FN	Se	Value of *p*	TP	FN	Se	Value of *p*	TP	FN	Se	Value of *p*	TP	FN	Se	Value of *p*
Test	85	11	88.5	<0.0001	117	11	91.4	<0.0001	44	4	91.7	0.0528	246	26	90.4	<0.0001
Control	19	77	19.8		77	51	60.2		36	12	75.0		132	140	48.5	
	TN	FP	Sp		TN	FP	Sp		TN	FP	Sp		TN	FP	Sp	
Test	15	1	93.8	0.0021	32	0	100.0	>0.9999	31	1	96.7	>0.9999	78	2	97.5	0.0174
Control	6	10	37.8		32	0	100.0		31	1	96.9		69	11	86.3	

TP, true positives; FN, false négatives; TN, true negatives; FP, false positives; Se, sensitivity; Sp, specificity. Value of *p*s correspond to Fisher’s exact tests.

### 3.3 Questionnaires

Although the theoretical and practical ability ratings were similar on the initial questionnaire, the practical ability rating was higher in the test than in the control groups (Supplementary Table S1). Confidence scores in the clinical examination were not different between the two groups at both 1 and 6 months. We also noted that despite the initial balance between the two groups in terms of professional experience, theoretical knowledge scores were higher in the control group at inclusion, and a reversal of the trend was observed at 1 month with balanced scores at 6 months. Training with the simulator did not seem to influence the imaging prescriptions (Supplementary Table S1 and Supplementary Data).

## 4 Discussion

The present study revealed that VR simulation-based training with VertImage led to an optimization of diagnostic performances in emergency physicians using the HINTS method for the detection of central or peripheral origin of an acute vestibular syndrome.

VR simulation tools in pedagogic domains have been developed since the 80s and represent a great advance in terms of education (24, 25). Indeed, the association of simulation with a theoretical lesson has shown its superiority to a conventional theoretical class (26–28). Video contents are still interesting but insufficient to reflect several aspects of the clinical field such as eye-hand coordination, self-positioning, and ergonomics. As demonstrated by Sarmah et al. (29), low- and high-fidelity simulators are useful for improving clinical skills. Similar to technical skill simulators which are frequently employed in surgery (19, 21, 30), clinical examination simulators are at present the major formation tool for physicians to approach real-life situations since training in many life-threatening pathologies is limited or impossible. However, these tools do not exempt the presence of a supervisor during the training sessions (16). In the future, artificial intelligence may in part replace the supervision during training.

Our simulator offered the crucial possibility of multiple examinations of the same case by the trainee and variations of the scenario with different difficulty levels. Several aspects can still be improved: Haptic and visual feedback with a realistic skin texture and a variable cervical stiffness, which is an important factor in the elderly, might enhance the immersion rate, and a higher sampling rate and a lower system latency could also improve the experience for the perception of rapid ocular movements. As with all simulators, motion smoothness is correlated to software and GPU performance, and advances in this field will further optimize the system in the near future.

Acceptance and familiarization of the participants are also important parameters when facing continuously evolving technologies. However, as long as time is allowed to discover the simulator for both trainers and learners, there seems to be no significant impact of unawareness of new technologies on the training (31).

In our study, we evaluated the physicians on the same system used for their training. This method provided us with indications on the retention of theoretical and practical capacities. To evaluate the real-life performances, it would have been interesting to follow a group of emergency physicians and their patients for several months from the first visit for an acute vestibular syndrome to the final diagnosis. Another possibility would be to evaluate HIT performances in trained and novice practitioners on real patients and validate the results by video recordings of the HIT (vHIT). Generally, performances on a simulator and in the field are hard to correlate. Indeed, only a few studies compare both sides of the apprenticeship because following and evaluating participants in their daily exercise are difficult to set up. A valid follow-up is possible in surgery training (32–34) but almost impossible for emergency clinical evaluations or rare diseases.

Training on VertImage was associated with an improvement of self-assessed practical capacities 1 month after the session, but despite a higher diagnostic performance, there was still no progression of the ratings regarding confidence in the clinical examination after the VR training. The reason for this lack of confidence could be the absence of exposure to real-life situations requiring the HINTS method or insufficient training. These observations raise the issue of the training repetition and its frequency to obtain optimal retention. A recent study conducted by Anderson et al. (35) compared monthly versus quarterly formation for cardiopulmonary resuscitation. They showed that monthly training provided the highest performances in comparison to 3-, 6-, and 12-month training intervals during a 12-month period. Regular practice is frequently requested by the participants as self-confidence is acquired by repeated confrontations with the situation. A study on training in pediatric emergency care in 46 physicians and residents showed that all participants were in favor of repeating the training at a rate of 2 ± 1 sessions per year (36). However, frequent training sessions have a higher cost, and the optimal training frequency should be determined for each type of task and training scenario. This issue is the subject of a future work with our training system.

In addition to the observational and manual skills, the simulator-based approach appears to enhance the problem-solving capacities in similar domains (26). In the case of dizzy patients, VR-based methods will potentially enhance the interest of the trained practitioners to develop their theoretical and practical knowledge in dizziness beyond the HINTS method. In this context, the possibility to create different and individualized scenarios is of great importance, as focusing on specific points during the training improves self-confidence and allows one to confront similar situations with less stress than in real-life situations (37). Moreover, studies on the evolution of diagnostic and therapeutic skills over time after a simulation-based training session show that personalized protocols tend to elicit a longer retention (30, 38, 39). With VertImage, numerous combinations of clinical features can be customized, and difficulty levels can be adapted to the trainee. The effect of this personalization is also an interesting subject to assess.

The intervention of a trainer is also a crucial point in the outcome. The role of the trainer is also important to analyze: Does our training require an expert or can it be replaced by an explicative video? Would it be interesting to integrate the trainer’s suggestions into the virtual scene? These questions underline the value of an expert in reinforcing fieldwork knowledge and self-confidence. More focused studies are needed to determine the specific pedagogic features that influence the training outcome.

Another advantage of simulators such as VertImage is that they can serve as a basis for interprofessional education through the development of reflective practitioners and the creation of relevant learning experiences in small groups (40, 41).

The use of virtual reality simulation-based tools in the medical field can be enriched by the expertise in other fields such as the aviation industry or the army (42). These domains have developed, used, and validated VR-based simulators for the professional curriculum. The most prominent common aspects between these fields and medicine are the safety and the human factor in decision-making and conducting procedures. Regarding the human factor, managing uncertainty in routine conditions is another relevant issue in the emergency department. This point is by itself an entire field of study well described by Uri Hasson (43) who developed the theory of statistical learning similar to artificial intelligence. In this optics, it is important to assess potential influencing factors of uncertainty management such as supervision, reinforcement, repetition, and variation of situations, and its relationship with self-confidence.

The limitations of this study are important to underline. First, we conducted a single-center study with only one emergency department’s habits and training, which could impede an adequate generalization. The sample size was small (34 physicians), but all grade levels were represented. A potential selection bias could be raised following the division of the participants into two groups but was limited by randomization.

The use of an artificial tool is also one of the limitations as it will never be as real as the physical examination of humans. The software has fixed parameters that will potentially be improved with new technologies, such as better flow of the movements and more realistic haptic feedback or new options. In our study, we limited skew deviation with one eye moving during the cross-cover test for training facilities, but training with both eyes moving in opposite directions could be set up.

## 5 Conclusion

A mannequin-based VR tool (VertImage) was developed to immerse emergency physicians in front of a virtual dizzy patient and train them for the HINTS method. In combination with a theoretical course, this tool showed a significant advantage in terms of diagnostic performance over the theoretical course. Physicians could distinguish peripheral from central-type vertigos with higher sensitivity and specificity after the VR training. The follow-up showed a good retention over time. VR-trained physicians kept the advantage of higher diagnostic performance 6 months after the session. Despite greater diagnostic performances, self-confidence in the clinical examination was not influenced by the VR training. Training repetition and on-site coaching seem to be necessary to enhance self-confidence.

## Data availability statement

The original contributions presented in the study are included in the article/Supplementary material, further inquiries can be directed to the corresponding authors.

## Ethics statement

The studies involving humans were approved by Délégation à la Recherche Clinique et à l’Innovation (DRCI), CHU Dijon, France. The studies were conducted in accordance with the local legislation and institutional requirements. The participants provided their written informed consent to participate in this study. Written informed consent was obtained from the individual(s) for the publication of any potentially identifiable images or data included in this article.

## Author contributions

GU: Conceptualization, Data curation, Formal analysis, Investigation, Methodology, Resources, Writing – original draft. MC: Conceptualization, Data curation, Investigation, Methodology, Resources, Visualization, Writing – original draft, Writing – review & editing. JR: Software, Writing – review & editing. ChG: Software, Writing – review & editing. CaG: Conceptualization, Visualization, Writing – review & editing. CT: Conceptualization, Methodology, Supervision, Validation, Writing – review & editing. AB: Conceptualization, Formal Analysis, Funding acquisition, Methodology, Project administration, Resources, Supervision, Validation, Writing – review & editing.
