# Supplementary material for: Virtual-reality-enhanced mannequin to train emergency physicians to examine dizzy patients using the HINTS method
Source: Front Neurol. 2024 Jan 5;14:1335121. doi: 10.3389/fneur.2023.1335121 (PMC10796789; doi:10.3389/fneur.2023.1335121)
Supplement: Supplementary file 1 [file Table_1.docx]

**Supplementary Table 1** – Questionnaire results on daily use of HINTS protocol. Two physicians were lost to follow-up at 6 months. * p< 0.05 et ns: not significant, versus test group, non-parametric Mann-Whitney test for continuous and exact Fisher test for categorical variables.

|  | Test (Simulator) | Control |
| --- | --- | --- |
| **Inclusion (n=17 in each group)** |  |  |
| HINTS knowledge (% of yes) | 76% | 71% |
| HINTS practice (% of yes) | 24% | 53% |
| Theoretical knowledge (score /10) | 4.5 ± 1.81 [3-9] | 5.7 ± 1.57 [3-8] * |
| Practical ability (score/10) | 4.3 ± 1.99 [2-9] | 5.29 ± 1.79 [3-8] ns |
| Self-confidence (score/10) | 4.4 ± 2.03 [3-8] | 5.2 ± 2.48 [3-8] ns |
| Imaging prescription in all dizzy patients | Always: 0  > 50%: 41%  < 50%: 53%  Never: 6% | Always: 0 ns  > 50%: 53%  < 50%: 47%  Never: 0 |
| Imaging prescription in peripheral vertigo | Always: 0  Depending on context: 82%  Never: 18% | Always: 0  Depending on context: 47%  Never: 53% |
| **1 month (n=17 in each group)** |  |  |
| Theoretical knowledge (score /10) | 6.8 ± 1.47 [3-9] | 5.9 ± 1.58 [3-9] ns |
| Practical ability (score/10) | 6.5 ± 1.50 [3-8] | 5.2 ± 2.21 [2-9] * |
| Self-confidence (score/10) | 6.7 ± 1.62 [3-9] | 5.3 ± 2.26 [2-9] ns |
| Imaging prescription in all dizzy patients  (n=11 test, n=6 control) | Always: 0  > 50%: 28%  < 50%: 54%  Never: 18% | Always: 0 ns  > 50%: 33%  < 50%: 50%  Never: 17% |
| Imaging prescription in peripheral vertigo  (n=9 test, n=6 for control) | Always: 0  Depending on context: 44%  Never: 66% | Always: 0 ns  Depending on context: 67%  Never: 33% |
| **6 months (n=16 in each group)** |  |  |
| Use of HINTS method during the last 6 months | > 50%: 6%  < 50%: 6%  Never: 6%  No dizzy patient: 25% | > 50%: 25% ns  < 50%: 19%  Never: 0%  No dizzy patient: 19% |
| Theoretical knowledge (score/10) | 7.00 ± 1.41 [5-8] | 6.4 ±1.36 [3-9] ns |
| Practical knowledge (score/10) | 7.00 ± 1.41 [5-9] | 6.31 ± 1.35 [3-9] ns |
| Self-confidence (score/10) | 6.75 ± 1.88 [2-10] | 6.56 ± 1.46 [3-9] ns |
| Imaging prescription in all dizzy patients  (n=14 both groups) | Always: 0  > 50%: 21%  < 50%: 72%  Never: 7% | Always: 0 ns  > 50%: 21%  < 50%: 72%  Never: 7% |
| Imaging prescription in peripheral vertigo  (n=13 test, n=12 control) | Always: 15%  Depending on context: 54%  Never: 31% | Always: 0 ns  Depending on context: 58%  Never: 42% |
