# Supplementary material for: Virtual-reality-enhanced mannequin to train emergency physicians to examine dizzy patients using the HINTS method
Source: Front Neurol. 2024 Jan 5;14:1335121. doi: 10.3389/fneur.2023.1335121 (PMC10796789; doi:10.3389/fneur.2023.1335121)
Supplement: Supplementary file 2 [file Data_Sheet_1.docx]

Supplementary Data – Trainer interface: Skew deviation (B) and Head Impulse Test (C) analysis.
